# Periodontitis as a Preterm Birth Risk Factor in Caucasian Women: A Cohort Study

**DOI:** 10.3290/j.ohpd.a44116

**Published:** 2020-04-01

**Authors:** Leticia Caneiro, Jose Manuel Lopez-Carral, Pablo Martin-Lancharro, Antonio Linares, Pilar Batalla, Juan Blanco-Carrion

**Affiliations:** a Researcher, Department of Estomatology, University of Santiago de Compostela, Santiago de Compostela, Spain. Conceptualisation, data curation, investigation, writing manuscript (original draft).; b Researcher, Department of Obstetrics and Gynaecology, Clinic University Hospital, Santiago de Compostela, Spain. Methodology, resources.; c Researcher, Clinic University Hospital, Santiago de Compostela, Spain. Data curation, formal analysis.; d Assistant Professor, Department of Estomatology, University of Santiago de Compostela, Santiago de Compostela, Spain. Visualisation, writing, review and editing (final manuscript).; e Assistant Professor, Department of Estomatology, University of Santiago de Compostela, Santiago de Compostela, Spain. Supervision, writing, review and editing (final manuscript).; f Professor, Department of Estomatology, University of Santiago de Compostela, Santiago de Compostela, Spain. Conceptualisation, funding acquisition, methodology, project administration.

**Keywords:** preterm birth, periodontitis, pregnancy, risk factor

## Abstract

**Purpose:**

The aim of this study was to evaluate the association between periodontitis and preterm birth in a Spanish Caucasian population, based on clinical and biochemical outcomes. Epidemiological studies have suggested that periodontitis is a potential risk factor for preterm birth. However, other studies have shown high heterogeneity in their results. Some factors such as number of evaluations during pregnancy, sample size, study population and maternal age may have an impact on the variability of the result.

**Methods and Materials:**

This cohort study enrolled 158 pregnant women, 39 with periodontitis and 119 without periodontitis. All pregnant women were evaluated in the first, second and third trimester.

**Results:**

Statistically significant differences were found in periodontal parameters between both groups, but no statistically significant differences were found in biochemical parameters during pregnancy. The duration of pregnancy in healthy patients was 38.78 ± 4.49 weeks, and in patients with periodontitis 37.81 ± 4.89 weeks, with no statistical difference (p > 0.05). This showed that periodontitis was not associated with preterm birth in a Spanish Caucasian cohort.

**Conclusion:**

In this study, periodontitis stage II, grade B, was not statistically associated with preterm birth. Pregnancy is a short period of time in order to evaluate long-term oral systemic infections. Adverse pregnancy outcomes are more difficult to occur. Thus, since pregnancy timing average cannot be changed, the stages of periodontal disease (initial, moderate, advanced) could be another factor to study.

Multiple factors have been associated with preterm birth-low birth weight (PLBW) and some authors have emphasised the heterogeneity of the causes.^12^ Among the known risk factors are: young maternal age, low maternal weight gain, low pregravid weight, multiple gestations, gestational diabetes, genitourinary tract infections, drug use, cigarette smoking and excessive alcohol consumption, while previous preterm delivery is a strong predictive marker of future preterm labor.^6^ However, a statistically significant proportion of PLBW is still of unknown aetiology.

Periodontal diseases are a group of infectious diseases caused by predominantly Gram-negative and anaerobic bacteria that induce local and systemic elevations of pro-inflammatory prostaglandins (PGE2) and cytokines.^26^

A few years ago, it has been postulated that distant infections like periodontal diseases may be associated with PLBW through infection.^23^ Furthermore, there is ample evidence that periodontal bacteria frequently enter inside the bloodstream.^1^ The relationship between periodontal diseases and various systemic diseases was thought to be unidirectional. Currently, there is increasing evidence that the relationship between some of these entities may be bidirectional.^5^

The hypothesis linking subclinical infection and PLBW suggests that microbes themselves or microbial toxins, such as endotoxins, enter the uterine cavity during pregnancy by the ascending route from the lower genital tract, or by the blood-borne route from a non-genital focus. Periodontal infections may mediate PLBW through one or more of the following mechanism: (1) contamination of the fetoplacental unit by periodontal pathogens; (2) effects of lipopolysaccharide (LPS) from the periodontal reservoir on the fetoplacental unit; and (3) effects of the inflammatory mediators (ILs, prostaglandins and TNF-α) from the periodontal reservoir on the fetoplacental unit.^16^

Some studies were carried out on predominantly African-American, low-income level and younger groups of women. These are variables that are related with low birth weight (LBW)/preterm birth (PB) and PLBW.^6^

There are several risk factors common to both periodontal diseases and adverse pregnancy outcome. These include non-white ethnicity, socioeconomic factors and tobacco smoking.^9,10^

In attempting to account for this wide variance in risk estimates, one theory that arises is that the observed association is linked to the confounding effects of risk factors other than periodontal infection.^28^ This theory is supported by the fact that similar studies have yielded conflicting results,^22^ which are still being reported in recent publications.^5,21^

Therefore, this association must be further explored in observational and interventional studies, to establish whether it is causal in nature or incidental, and to determine the possible benefits of intervention and the potential to generalise the findings in diverse populations.^17^

The aim of this study was to evaluate the association between periodontitis and PB in a Spanish Caucasian pregnant women cohort based on clinical and biochemical outcomes.

## Material and Methods

### Study Population and Study Design

A prospective cohort study was designed to evaluate the association between PB and/or LBW and periodontitis. PB was defined as a delivery at < 37 weeks of gestation (gestational age determined by last menstrual period and ultrasound fetal measurement). LBW was defined as 2500 g or less newborn weight (World Health Organization). This cohort study was done in accordance with the Declaration of Helsinki, following STROBE guidelines and approved by the Ethics Investigation Committee of Santiago-Lugo (2016/451).

Between January 2016 and March 2017 pregnant women, who were seen for prenatal care in their first gynaecological clinical appointment (University Hospital of Santiago de Compostela, Spain), were invited to participate in the study. Pregnant patients were divided in two groups: case group corresponds to patients with periodontitis and control group were patients without periodontitis (healthy). Participants signed the informed consent statement to participate in the study.

The inclusion criteria were: Caucasian women aged 18–40 years; gestational age ≤16 weeks; more than 20 natural teeth.

Exclusion criteria were: multiple gestation; previous PLBW; more than two previous miscarriages or one after 18 weeks; history of diabetes; hypertension; alcoholism; drug abuse; human immunodeficiency virus (HIV); heart disease, kidney disease or liver disease; cystitis recurrence; viral infections; venereal infections; toxoplasmosis.

Demographic and medical history data were obtained through an interview during the first visit (16th gestational week). The variables included were age, educational level (primary or less, high school and university), residence (rural or urban, established based on the number of inhabitants), smoking habits (never smoked or former smoker before pregnancy, former smoker of < 15 cigarettes/day just until pregnancy, smoker of ≥15 cigarettes just until pregnancy, smoker < 6 cigarettes during pregnancy and smoker of ≥6 cigarettes during pregnancy), and obstetric history (number of previous pregnancies, previous miscarriage).

The following variables were recorded shortly after delivery: newborn weight, newborn sex, pregnancy time and type of delivery (vaginal or caesarean).

### Experimental Design

Data were obtained at the three usual follow-up pregnancy visits: end of the first trimester (16 weeks of pregnancy); at the middle of second trimester (23–25 weeks of pregnancy); and at the middle of the third trimester (33–36 weeks of pregnancy). Maternal characteristics were registered in the first visit and periodontal and biochemical data were obtained in each of the three visits. After delivery, periodontal treatment was performed in patients with periodontitis.

### Periodontal Measurements

Full-mouth periodontal exams were performed at enrolment and repeated at the 2nd and 3rd trimesters at the University Hospital of Santiago de Compostela (Spain). Exams included all teeth present in mouth (excluding third molars). A plaque score,^25^ periodontal pocket depth (six sites per tooth), clinical attachment level (CAL) and bleeding on probing (BOP) (six sites per tooth) were registered. All scores were measured with a manual periodontal probe UNC-15 (Hu-Friedy, Chicago, IL, USA).

The examinations were conducted by the same dentist (LC) and the weighted қ values for intraexaminer calibration were 0.82 (confidence interval (CI) 95% ± 0.68–0.98). Periodontal disease was defined according to the case definitions of the World Workshop in Periodontology in 2017^27^:

1. Interdental CAL was detectable at ≥ 2 non-adjacent teeth, or
2. Buccal or oral CAL ≥ 3 mm with pocketing ≥ 3 mm was detectable at ≥ 2 teeth but the observed CAL could not be ascribed to non-periodontitis-related causes such as: (1) gingival recession of traumatic origin; (2) dental caries extending in the cervical area of the tooth; (3) presence of CAL on the distal aspect of a second molar and associated with malposition or extraction of a third molar; (4) an endodontic lesion draining through the marginal periodontium; and (5) the occurrence of a vertical root fracture.^27^

### Biochemical Variables

Peripheral blood sample was collected from each subject with venipuncture using a vacuum tube (Vacutainer, Nippon Becton Dickinson, Tokyo, Japan). Samples were centrifuged at 2500 rpm for 10 min and the serum obtained was dissociated in a plastic tube. At the time of the analyses, the serum concentration of interleukin-6 (IL-6), interleukin-8 (IL-8), interleukin-1β (IL-1β), tumour necrosis factor-α (TNF-α), c-reactive protein (CRP) and fibrinogen were determined using commercially available enzyme-linked immunoassays (ELISA). All biochemical analyses were performed in University Hospital of Santiago de Compostela (Spain).

### Statistical Analysis

Descriptive statistical analysis was calculated for each variable (for continuous variables, mean and standard deviations (SDs), and numbers and percentages were calculated for categorical variables). The Kolmogorov-Smirnov test was performed to evaluate the normality of the variables. The relationship between each variable and PLBW was analysed. All variables were also compared between women with and without periodontitis. Student’s t test was performed for continuous variables and Chi-square test (*X*^2^) was used to analyse categorical variables. Statistical significance was established at the 95% confidence level and p value < 0.05.

A multivariate logistic regression analyses was performed using the forward step method to assess risk factors associated with PLBW. Adjusted odds ratios (OR) with a 95% CI for PB risk were calculated. Power calculation was 80%, and p value < 0.05 for all analyses was selected to be statistically significant. SPSS for Windows (SPSS, version 20.0, IBM, NY, USA) was used for all data analyses.

## Results

**Fig 1:**
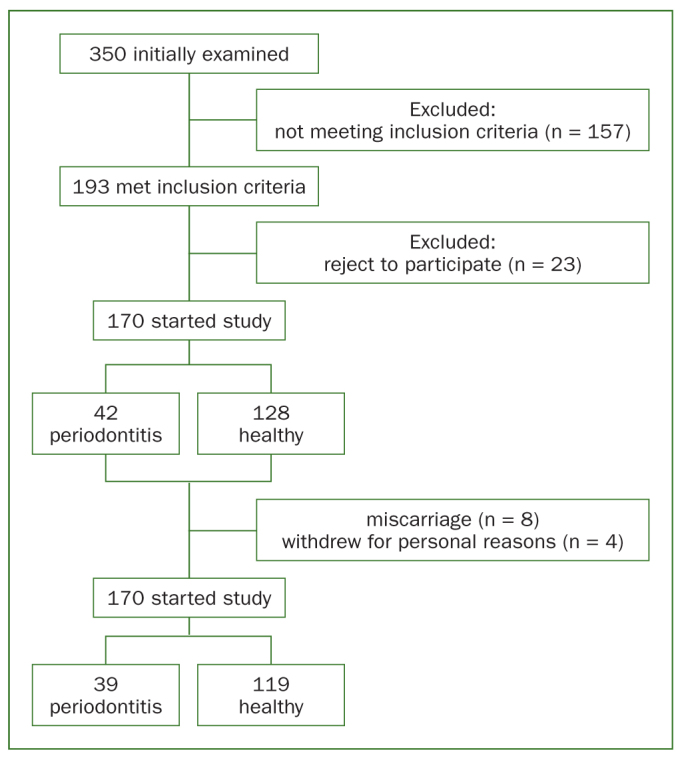
Flow chart of patients.

Initially 350 women were examined and 193 met the inclusion criteria. Twenty-three declined to participate, resulting in a final sample of 170 women. Periodontitis was present in 42 women and absent in the remaining 128; 8 of these women had a miscarriage before the second visit, and 4 withdrew after the second visit for personal reasons. Finally, 39 women with periodontitis stage II, grade B and 119 without periodontitis complied to all the visits (Fig 1).

Table 1 summarises demographic and social variables. All these variables showed no statistical differences meaning homogeneity between both groups.

**Table 1:** Demographic and social description of the study population

	Healthy group	Periodontitis group
**Age**	31.91 ± 4.21	31.88 ± 4.38
**Education level**		
Primary	67.188%	66.66%
Secondary	30.46%	28.57%
University	2.34%	4.76%
**Residence**		
Rural	92.18%	90.47%
Urban	7.81%	9.52%
**Smoking**		
Never or ex-smoker	78.90%	80.95%
Ex-smoker pregnancy < 15 cigarettes	13.28%	7.14%
Ex-smoker pregnancy ³ 15 cigarettes	1.56%	4.76%
Smoker < 6 cigarettes	4.68%	4.76%
Smoker ³ 6 cigarettes	1.56%	2.38%
**Marital status**		
Single	1.56%	2.38%
Common law partner	35.93%	40.47%
Married	62.50%	57.14%

All periodontal parameters showed statistically significant differences between the two groups in the three trimesters (p < 0.01 for all comparisons; see Table 2). The plaque index in healthy patients was maintained during the whole pregnancy with values below 20%, while patients with periodontitis had levels of plaque around 40%. BOP, clinical attachment loss ≥ 3 mm and percentage of probing depth ≥ 4 mm in patients with periodontitis was greater in the three trimesters. Healthy patients had more than 90% of probing depth ≤ 3 mm.

**Table 2:** Evolution of periodontal clinical parameters during visits

		1st visit	2nd visit	3rd visit
Number of teeth	Healthy	26.22 ± 2.18	26.22 ± 2.18	26.22 ± 2.18
	Periodontitis	26.67 ± 1.89	26.67 ± 1.89	26.67 ± 1.89
p value		0.235	0.235	0.235
Plaque index	Healthy	18.33 ± 11.56	18.44 ± 10.07	18.86 ± 10.33
	Periodontitis	40.36 ± 21.94	39.94 ± 20.00	29.79 ± 19.77
p value		< 0.01	< 0.01	< 0.01
Bleeding on probing	Healthy	14.34 ± 10.84	18.14 ± 11.87	17.29 ± 11.68
	Periodontitis	38.18 ± 14.32	47.67 ± 18.87	50.67 ± 30.99
p value		< 0.01	< 0.01	< 0.01
Probing depth ≤ 3 mm (%)	Healthy	94.08 ± 9.42	93.75 ± 9.67	94.03 ± 10.43
	Periodontitis	68.26 ± 16.91	60.52 ± 10.24	69.58 ± 19.25
p value		< 0.01	< 0.01	< 0.01
Probing depth ≥ 4 mm (%)	Healthy	5.91 ± 6.88	6.60 ± 6.80	6.24 ± 7.02
	Periodontitis	31.90 ± 15.82	40.13 ± 18.60	31.68 ± 19.36
p value		< 0.01	< 0.01	< 0.01
Clinical attachment loss ≥ 3 mm (%)	Healthy	0.86 ± 0.26	0.77 ± 0.33	0.81 ± 0.37
	Periodontitis	6.1 (1.3)	6.6 (1.6)	6.7 (1.4)
p value		< 0.01	< 0.01	< 0.01

Student’s t test for number of teeth, plaque index and bleeding on probing. c2 for probing depth ≤ 3 mm, probing depth ≥ 4 mm and clinical attachment loss ≥ 3 mm.

**Fig 2:**
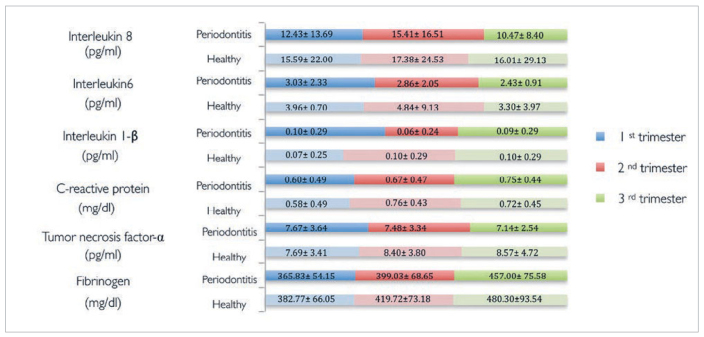
Evolution of biochemical parameters during pregnancy.

Values of fibrinogen and CRP experienced a slight increase over the three trimesters in both groups, but there was no statistical difference at any trimester. Values of tumour necrosis factor-α and interleukin-1β were kept stable during the three trimesters in periodontal patients, while in healthy patients these increased slightly. However, no statistical difference was observed between groups. Levels of interleukin-6 and interleukin-8 were higher in healthy patients, although no difference was found between groups throughout pregnancy (Fig 2).

Number of previous pregnancies was zero in 80 of healthy patients, 36 had one previous pregnancy and 3 had two previous pregnancies. Patients with periodontitis, 29 had no previous pregnancy, 10 had one previous pregnancy and none of them had two or more.

In the healthy group, 95 had no previous miscarriage, 23 patients had one previous miscarriage and 1 had two, whereas in the patients with periodontitis, 36 had no previous miscarriage, 3 patients had one previous miscarriage and no patients had two or more miscarriages. The number of miscarriages during pregnancy was 8 in total, 5 of which occurred in the healthy group and 3 in the periodontitis group.

Table 3 summarises obstetric history in relation to duration of pregnancy and newborn weight. Duration of pregnancy mean was greater than 37 weeks and weight mean of the newborn was higher than 2500 grams in both groups. There were no statistically significant result between both groups (Table 3).

**Table 3:** Obstetric history

Duration of pregnancy (weeks)	Healthy	38.78 ± 4.49	p = 0.731
	Periodontitis	37.81 ± 4.84	
Newborn weight (gram)	Healthy	3285.80 ± 457.59	p = 0.686
	Periodontitis	3146.15 ± 477.77	

Student’s t test for duration of pregnancy and newborn weight.

Multivariate analysis (logistic regression) was carried out three times (three visits) to gauge each risk factor influences within each phase, in order to assess risk factors (age, biochemical, smoking habits and periodontitis) associated with PLWB. Interleukin-6 was predictive but not statistically significant at each trimester (eg, fibrinogen was predictive but not statistically significant at 3º trimester). Only CRP was statistically significant at 1º trimester (CI 0.037–0.789). Periodontitis was not statistically significant at any trimester (Table 4).

**Table 4:** Multivariate logistic regression analyses

Logistic Regression										
Sig.		1º visit			2º visit			3º visit		
		Step 2 sig.	IC 95%	Sig.	Step 1 sig.	IC 95%	Sig.	Step 2 sig.	I.C. 95%	
Step 0	Fibrinogen	0.416			0.416			0.046	0.054	1.000–1.011
	C-reactive protein	0.011	0.024	0.037–0.789	0.495			0.102		
	Tumour necrosis factor-α	0.598			0.336			0.070		
	Interleukin-6 (IL-6)	0.017	0.102	0.988–1.145	0.031	0.062	0.998–1.092	0.035	0.073	0.991–1.224
	Interleukin-8 (IL-8)	0.991			0.867			0.866		
	Interleukin-1β (IL-1β)	0.906			0.109			0.250		
	Never or ex-smoker	0.544			0.426			0.674		
	Ex-smoker pregnancy < 15 cigarettes	0.881			0.411			0.525		
	Ex-smoker pregnancy > 15 cigarettes	0.789			0.389			0.167		
	Smoker < 6 cigarettes	0.539			1.000			0.600		
	Smoker > 6 cigarettes	0.318			0.614			0.561		
	Age	0.656			0.279			0.046		
	Periodontitis	0.183			0.149			0.174		

## Discussion

In this study, the relationship between periodontitis and PLWB was evaluated in a Caucasian pregnant cohort. This investigation was not able to show an association between periodontitis with PLWB. One possibility for this is that while the definitions of PB and LBW are well established, no consensus has yet been achieved on the definition of periodontitis in periodontal research, which is essential to optimise the interpretation, comparison and validation of clinical data.^4^

For the present study, the definition of periodontitis used was the one proposed in the last World Workshop of Periodontology.^27^ In the meta-analysis published by Ide et al,^12^ the authors showed that the commonly used periodontitis definitions by López et al^14,15^ resulted in statistically significant positive associations between maternal periodontitis and adverse pregnancy outcomes in study population, but in contrast, analyses of data from the same populations using mean probing depths and other continuous variables frequently resulted in non-statistically significant associations.

Seven cohort studies found association between PB/PLBW^3,13,14,15,17,21,24^ and periodontitis; however, three cohort studies found no association^7,18,20^; these cohort studies varied in sample size, type of population, presence and management of aetiologic or risk factors and maternal age.

In our study maternal age has not been a risk factor for PB/LBW (mean age was 31.68 years). This result was contrary to other investigations which have used samples composed of younger women.^19^

A statistically significant association between periodontitis and adverse pregnancy outcomes has been found in populations with a high incidence of preterm deliveries, including American-African women and those from economically disadvantaged families.^3,13-15,23,24^ In contrast, most studies conducted in European countries or Canada, which offer universal health care, have shown significantly lower percentages of PB and/or LBW and no association between periodontitis and adverse pregnancy outcomes.^7,18,20^

In previous studies published in the literature, an increase in maternal serum levels of pro-inflammatory markers such as TNF-α, IL-6, IL-8, IL1-β and CRP may be associated with PB/LBW.^11,29^ In our study, this association was only found in CRP at first trimester. A potential explanation may be related to the use of serum samples instead of crevicular fluid. However, there is limited and negative evidence that the elevations of these mediators in gingival crevicular fluid, serum and amniotic fluid are associated with pregnancy complications in periodontitis patients.^8^

One limitation of our study was the number of patients with periodontitis. This represents only a 25% of the total population. Another limitation was the severity of the disease. The use of the new classification may well help to establish different degrees of staging/grades and risk to PB/LBW.

## Conclusion

In conclusion, the present investigation showed that periodontitis stage II, grade B, does not increased the risk of preterm birth/LBW in our study population. However, there was a 1-week delivery and a 140 g weight difference between the groups that could be amplified with a larger and more advanced periodontitis group. Thus, the association between periodontitis and PB should be further explored in intervention studies to establish whether it is causal or incidental.
